# Targeting Quorum Sensing to Combat Foodborne Pathogens: A Dual Strategy Against Spoilage and Pathogenesis

**DOI:** 10.3390/foods15142439

**Published:** 2026-07-09

**Authors:** Chen Niu, Jing Yang, Chaofan Kong, Rui Cai, Yahong Yuan, Tianli Yue

**Affiliations:** 1 College of Food Science and Technology, Northwest University, Xi’an 710069, China; 2 Laboratory of Nutritional and Healthy Food-Individuation Manufacturing Engineering, Xi’an 710069, China; 3 Research Center of Food Safety Risk Assessment and Control, Xi’an 710069, China

**Keywords:** quorum sensing, foodborne bacteria, food spoilage, pathogenesis, intervention

## Abstract

Foodborne pathogens rely on colonization, biofilm formation, virulence expression, and environmental adaptation as fundamental biological drivers of food safety risk. Quorum sensing (QS), a cell-density-dependent microbial communication mechanism, coordinates the expression of these key phenotypes by integrating intraspecies, interspecies, and host-derived signals, making QS an attractive intervention target in food microbial control. Although QS research has advanced considerably in recent years, existing reviews have largely focused on individual bacterial species or specific classes of signal molecules. A systematic integration of how QS coordinately drives both food spoilage and pathogen virulence remains lacking. In this review, we conceptualize the QS network as a central regulatory hub connecting microbial signal perception to hazardous phenotype expression. We systematically examine the mechanistic roles of QS in food spoilage, biofilm formation, host colonization and invasion, and toxin production. We also summarize current QS-targeted intervention strategies, including inhibition of signal synthesis, enzymatic signal degradation, receptor antagonism, and indirect regulation via beneficial microorganisms. Building on the available evidence, we further analyze the key challenges limiting practical application: signal system specificity, ecological safety, industrial-scale feasibility, and microbial adaptability. Overall, QS-based strategies offer a non-bactericidal route for food microbial control, although substantial barriers remain for translation into complex food matrices. Reframing QS function and intervention from the perspective of food safety risk formation provides an analytical framework that bridges mechanistic understanding with practical application. This framework also establishes a theoretical foundation for developing next-generation food preservation and foodborne disease control strategies.

## 1. Introduction

Currently, global food security is facing complex and severe challenges. The core issue lies in the persistently high number of hungry people worldwide and the significant losses within the food supply chain. According to the report by the Food and Agriculture Organization (FAO), the number of people facing hunger worldwide reached 733 million in 2023. However, at the same time, the phenomenon of global food waste is extremely serious, with a large amount of food being lost at various stages of the supply chain. Data shows that in 2022, approximately 1.05 billion tons of food were wasted, accounting for 19% of the total food available for consumers that year [1]. Food contamination, as one of the core causes of food loss, not only exacerbates food supply pressures but also directly threatens public health. According to the World Health Organization (WHO), nearly 600 million people suffer from foodborne diseases each year due to consuming contaminated food [2].

Among the various factors that cause foodborne diseases, bacterial contamination is the most widespread pathogenic cause [3]. Foodborne bacteria can impair food quality through extracellular hydrolytic enzymes and spoilage metabolites, while pathogenic strains may additionally threaten consumer health through toxin production and virulence factor expression, and can cause infectious or toxigenic disease via intestinal colonization, tissue invasion, and virulence factor expression [4,5,6,7]. In addition, many pathogenic bacteria form biofilms on food surfaces, processing equipment, and production environments, thereby enhancing their environmental tolerance and elevating the risk of persistent contamination [8]. Elucidating the key regulatory mechanisms governing these phenotypes is therefore essential for advancing food preservation strategies and foodborne disease control.

Quorum sensing (QS) is a widespread cell-to-cell communication mechanism in bacteria. Bacteria continuously synthesize, release, and sense signaling molecules termed autoinducers, thereby monitoring local population density and environmental dynamics in real time. When extracellular autoinducer concentrations reach a threshold, cognate receptors are activated, triggering the coordinated expression of specific gene clusters and enabling population-level behavioral regulation [9]. In contrast to the independent responses of individual cells, QS allows bacteria to integrate environmental information and synchronize physiological activities at the community level. It is therefore regarded as a central regulatory hub linking microbial individual behavior to collective phenotypes.

A growing body of evidence has demonstrated that QS participates extensively in the adaptation of foodborne pathogens within both food systems and host environments. QS regulates key phenotypes, including biofilm formation, motility, extracellular enzyme secretion, and virulence factor expression, and also contributes to the production of spoilage-associated factors such as proteases, lipases, biogenic amines, and volatile metabolites [10,11]. By coordinating these processes, QS facilitates the colonization and dissemination of spoilage organisms in food matrices while simultaneously enhancing pathogen survival, invasion, and virulence within the host. QS has therefore emerged as an important intervention target in food microbial control. Unlike conventional bactericidal strategies, QS-targeted interventions primarily disrupt signal molecule production, transmission, or recognition to attenuate bacterial collective behavior without directly inhibiting cell growth. This approach offers a novel technical pathway for food preservation and foodborne disease control.

Existing reviews have predominantly focused on specific signal molecule classes (e.g., AHLs, AI-2, and AIPs), individual pathogens, or single intervention strategies [12,13,14]. These studies have helped elucidate the composition and regulatory mechanisms of distinct QS systems. However, because they are largely organized by signal type or pathogen species, they tend to discuss QS networks in isolation from food spoilage and pathogenesis processes, making it difficult to reveal the integrative function of QS in food safety risk formation. From an industrial perspective, the more pressing question is not the function of any single QS system per se, but rather how QS coordinately drives food spoilage and pathogen virulence, and whether these risks can be effectively mitigated by targeting QS. To date, a review framework that systematically integrates QS mechanisms and intervention strategies from the perspective of spoilage- and pathogenesis-associated phenotypes remains absent.

In this review, we adopt a food safety risk formation perspective to systematically summarize the information transmission and functional integration mechanisms of QS networks in foodborne pathogens. We focus on the regulatory roles of QS in food spoilage and pathogenesis, and further synthesize current QS-targeted control strategies, their advantages, limitations, and future directions. This review aims to provide a theoretical foundation and reference framework for food preservation and foodborne disease control.

## 2. Multi-Level Communication Networks of QS in Foodborne Pathogens

QS is an essential regulatory mechanism by which bacteria perceive population density and coordinate collective behaviors. From a functional perspective, distinct QS systems do not operate in isolation; rather, they collectively constitute a multi-level information network that integrates intraspecies communication, interspecies interactions, and host adaptation. For foodborne pathogens, this networked organization enables rapid integration of diverse external signals within complex food environments and host milieus, thereby coordinating critical physiological processes including biofilm formation, virulence expression, environmental adaptation, and colonization and invasion. Understanding QS systems as a multi-level information network can therefore help reveal how environmental perception is translated into spoilage- and pathogenesis-associated phenotypes.

### 2.1. Species-Specific Signals Drive Collective Behavioral Coordination

**Figure 1:**
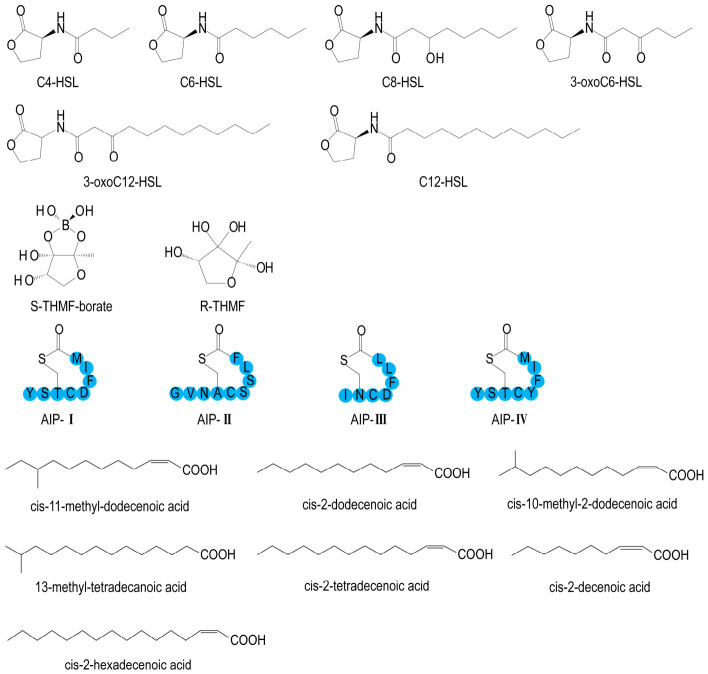
The chemical structure of signal molecules in QS systems.

Intraspecies communication represents the most classical functional modality of QS. Its defining feature is the use of species-specific signals to enable information sharing and behavioral synchronization among cells of the same species. Key examples include acyl-homoserine lactones (AHLs), diffusible signal factor (DSF), and autoinducing peptides (AIPs) (Figure 1). Gram-negative bacteria primarily rely on AHL-based systems [15], whereas Gram-positive bacteria predominantly utilize AIPs to mediate population-level communication [16].

The AHL-mediated LuxI/LuxR system is widely distributed among food-associated bacteria, including *Pseudomonas* spp., *Aeromonas* spp., and *Hafnia* spp. LuxI-family proteins are responsible for synthesizing AHL signal molecules, which consist of a hydrophilic homoserine lactone (HSL) ring and a variable hydrophobic acyl side chain (Figure 1). As cell density increases, extracellular AHLs progressively accumulate. Upon reaching a threshold concentration, AHLs bind to LuxR-type transcriptional regulators, thereby activating the expression of downstream target genes [15,17]. This system can coordinately regulate diverse collective behaviors, including biofilm formation, motility, extracellular enzyme secretion, and virulence factor expression.

A similar “signal accumulation–threshold sensing–population response” logic operates in Gram-positive bacteria via the AIP system. The accessory gene regulator (Agr) system of *Staphylococcus aureus* is a prototypical example of AIP-mediated intraspecies communication, coordinating virulence factor expression and the synchronization of collective behaviors [18].

Although AHLs and AIPs differ markedly in signal molecular structure and receptor recognition mechanisms, they serve a shared biological objective: achieving population-level behavioral synchronization through highly specific information exchange. This coordinated regulation enables bacteria to express virulence factors in concert, form robust biofilms, or initiate collective migration at opportune moments, thereby substantially enhancing environmental fitness and competitive advantage.

### 2.2. Universal Signal Systems Facilitate Interspecies Information Exchange

Real food systems are typically inhabited by multiple microbial species. Consequently, beyond intraspecies communication, extensive interspecies information exchange also occurs among community members. Among these cross-species signaling mechanisms, the AI-2 system is regarded as one of the most important.

AI-2 is derived from LuxS-mediated activated methyl cycle metabolism. Its precursor, 4,5-dihydroxy-2,3-pentanedione (DPD), can spontaneously form multiple structural isomers that are recognized by different bacterial species [19,20] (Figure 1). Unlike AHLs and AIPs, AI-2 is widely distributed across both Gram-negative and Gram-positive bacteria and is therefore considered a QS signal with “universal language” characteristics [21,22,23]. A large body of evidence indicates that AI-2 participates in regulating biofilm formation, host colonization, virulence expression, and metabolic adaptation [24,25].

From a food safety perspective, the importance of AI-2 lies not only in its ability to regulate the behavior of individual species, but more critically in its capacity to connect information networks across different species. In mixed-community environments, AI-2 released by one bacterial species can be sensed and exploited by another, thereby influencing community composition and collective behavior. This cross-species communication mechanism contributes to the formation of more stable and complex microbial communities while simultaneously increasing the difficulty of food spoilage control.

However, whether AI-2 truly qualifies as a QS signal molecule remains contentious. Because its precursor DPD may be a metabolic byproduct [26], some researchers argue that AI-2 is more likely a metabolic byproduct that bacteria have “co-opted” as a communication signal [27,28,29]. Furthermore, even among the AI-2 receptors identified to date, signal transduction mechanisms exhibit considerable diversity. The *Vibrio* LuxP receptor and the *Salmonella* LsrB transporter provide illustrative examples. Upon AI-2 binding, LuxP activates autophosphorylation of the membrane kinase LuxQ, triggering the LuxQ–LuxU–LuxO phosphorylation cascade to regulate virulence genes [30]. In contrast, LsrB internalizes AI-2 into the cell, where phosphorylation by the LsrK kinase enables the resulting phospho-AI-2 to bind the LsrR repressor, thereby relieving transcriptional repression of the *lsr* operon and initiating virulence gene expression [31]. This controversy reflects the incomplete consensus on the fundamental nature of QS and suggests that future research should more clearly delineate the boundary between signaling and metabolic functions.

### 2.3. Host-Associated Signals Mediate Interkingdom Information Exchange

Beyond intraspecies and interspecies communication, certain foodborne pathogens can also sense host-derived signals through QS systems, enabling interkingdom signaling. This capability extends the QS network beyond the conventional scope of bacterial information exchange, incorporating the host environment into the signal perception system. Consequently, pathogens can more accurately gauge their ecological niche and the optimal timing for infection.

The AI-3/QseC-associated system is currently the most extensively studied representative of this signaling modality. QseC can recognize host-derived catecholamine hormones, such as epinephrine and norepinephrine, and integrate these environmental cues into the cellular regulatory network [32]. Through this mechanism, pathogens can simultaneously monitor changes in population density and host physiological status, thereby achieving more precise environmental responses.

At the functional level, the significance of host-associated signaling systems extends beyond mere signal recognition. These systems establish a direct link between the host environment and pathogen behavior. When shifts occur in host physiological status, inflammatory state, or intestinal microenvironment, the corresponding signals can be perceived by pathogens and translated into regulatory instructions, thereby influencing their survival strategies and adaptive processes. Notably, accumulating evidence suggests that host-associated signaling systems may do more than merely “sense the host”—they may also participate in bidirectional information exchange between bacteria and host cells. For instance, AI-3 has been found to induce immune responses in host cells [33] indicating that certain QS signal molecules may simultaneously possess dual attributes of microbial communication and host modulation. Although the cognate receptors and underlying molecular mechanisms await further elucidation, these findings suggest that the QS network has transcended its classical role in bacterial population communication, progressively evolving into an information integration platform that connects microbial communities with the host environment.

### 2.4. From Information Communication to Food Safety Phenotypes

Although distinct QS systems differ markedly in signal molecular structure, receptor types, and signal transduction mechanisms, they do not function independently. Rather, they collectively form a multi-level information integration network. Intraspecies communication systems coordinate behavioral synchronization among cells of the same species; interspecies communication systems facilitate information exchange between different microbial community members; and host-associated signaling systems enable pathogens to sense changes in the host environment. By integrating information from these diverse sources, bacteria can mount more precise responses to external conditions.

Notably, the biological significance of the QS network extends beyond information transmission per se—its ultimate purpose lies in the regulation of specific functional phenotypes. Existing evidence indicates that although different QS systems receive distinct upstream signals, their downstream regulatory targets are highly overlapping, converging primarily on key processes such as biofilm formation, motility control, extracellular enzyme secretion, virulence factor expression, and environmental adaptation. These functional modules, in turn, determine the survival strategies of pathogens within both food systems and host environments.

From a food safety perspective, the functional outputs governed by the QS network largely manifest as two categories of outcomes. The first category promotes spoilage organism colonization, growth, and extracellular enzyme secretion, ultimately leading to food quality deterioration and reduced shelf life. The second category enhances the colonization, invasion, and toxigenic capacity of foodborne pathogens, culminating in foodborne disease. Understanding how the QS network drives these key functional phenotypes therefore represents a critical foundation for elucidating its mechanistic role in food spoilage and foodborne disease pathogenesis.

Building on this foundation, the following sections systematically discuss the regulatory roles of QS in the virulence and hazard-associated phenotypes of foodborne pathogens, spanning multiple dimensions including food spoilage, intestinal invasion, and toxin production.

## 3. QS-Mediated Food Spoilage and Pathogenic Processes

### 3.1. QS-Mediated Food Spoilage

#### 3.1.1. QS Promotes Spoilage Organism Colonization and Biofilm Formation

The initiation of spoilage depends on successful microbial colonization of food surfaces. QS enhances bacterial attachment and environmental fitness by regulating motility, biofilm formation, and collective migration behaviors. A substantial body of evidence indicates that AHL-mediated QS systems enhance flagellar motility, chemotaxis, and extracellular polymeric substance (EPS) synthesis, thereby promoting biofilm formation [34]. Biofilms serve as critical niches for aggregated bacterial growth and further enhance microbial tolerance to low-temperature storage, washing, and disinfection treatments [35]. Beyond providing physical protection for the microbial community, the EPS matrix produced within biofilms can directly cause surface sliminess and textural deterioration of food products [36]. Thus, QS-mediated regulation of biofilm formation and colonization capacity represents one of the essential prerequisites for spoilage onset.

#### 3.1.2. QS Promotes Spoilage Organism Exploitation of Food Matrix

The sustained proliferation of spoilage organisms in food systems depends on efficient acquisition and utilization of nutrients from the food matrix, a process under the regulatory control of QS systems. QS enhances bacterial utilization efficiency of complex macromolecular nutrients by regulating the production of extracellular hydrolytic enzymes such as proteases and lipases. Studies have confirmed that when AHL signal synthesis is inhibited in *Pseudomonas fluorescens*, the mRNA expression levels of protease genes (*prlC*, *ctpB*) and lipase genes (*lipA*, *lipB*) are significantly downregulated, ultimately reducing proteolytic and lipolytic activities [37]. In another study, exogenous C6-HSL signal molecules induced the upregulation of protease- and lipase-encoding genes (*lips*, *mucD*) in *Pseudomonas azotoformans*, thereby accelerating protein and lipid degradation [38]. Collectively, these lines of evidence indicate that QS functions as a direct regulatory switch governing hydrolase production and secretion.

#### 3.1.3. QS Regulates Spoilage Metabolites and Quality Deterioration

Following nutrient acquisition and sustained proliferation, spoilage organisms further produce a variety of spoilage-associated products through metabolic activities, leading to changes in sensory quality attributes including flavor, color, and texture. A growing body of evidence indicates that the formation of these spoilage phenotypes is also subject to significant regulation by QS systems.

In lipid-rich foods, QS-regulated lipase production promotes lipid hydrolysis and oxidation, generating volatile compounds such as aldehydes, ketones, and short-chain fatty acids that produce rancid and other off-odors [39]. In protein-rich meat and aquatic products, proteases secreted by spoilage organisms degrade macromolecular proteins into small peptides and amino acids, which are further converted into ammonia, amines, and sulfur-containing volatiles. This results in elevated total volatile basic nitrogen (TVB-N) levels and the generation of characteristic spoilage odors [40]. A growing body of evidence indicates that these metabolic processes are directly or indirectly regulated by QS systems. For example, a *luxR* deletion mutant of *Shewanella baltica*, disrupted in QS signal transduction, exhibited significantly lower growth rates and TVB-N production in refrigerated shrimp compared with the wild-type strain, markedly delaying the spoilage process [41]. Similarly, cadaverine production in a *luxR* mutant of *Hafnia alvei* was reduced by approximately 50% relative to the wild type [42]. Further investigation revealed that its AHL synthase LuxI promotes the synthesis of biogenic amines such as putrescine and cadaverine by regulating the expression of the ornithine carbamoyltransferase gene *argF* and the ornithine decarboxylase gene *speC* [40]. These findings demonstrate that QS not only influences nutrient degradation but also directly regulates the formation of spoilage metabolites.

Beyond flavor deterioration, QS also contributes to changes in food appearance quality. For instance, *Serratia marcescens* synthesizes pigments such as prodigiosin under QS regulation, leading to abnormal discoloration of food products [43]. Furthermore, in fruit and vegetable products, QS-regulated pectinases and cellulases degrade plant cell wall components, disrupting tissue structural integrity and causing textural deterioration such as softening and loss of crispness [10,11,44]. Collectively, QS-mediated regulation of spoilage metabolites and quality-deteriorating products ultimately manifests as declines across multiple sensory dimensions—flavor, color, and texture—thereby shortening product shelf life and reducing consumer acceptability.

**Figure 2:**
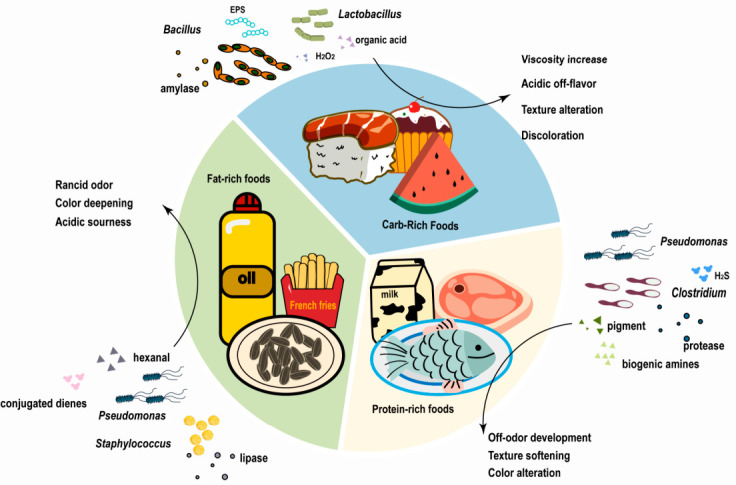
Food spoilage caused by bacteria.

Food spoilage primarily reflects the regulatory consequences of QS on microbial resource utilization and environmental adaptation, with its hazard largely manifested as food quality deterioration and economic loss (Figure 2). However, for foodborne pathogens, the functional outputs governed by QS are not confined to growth and metabolic activities within food matrices. QS further participates in colonization, invasion, and virulence expression within the host. At the food safety level, therefore, the scope of QS network action extends from food quality deterioration to the onset of foodborne disease.

### 3.2. QS-Mediated Host Colonization and Invasion

**Figure 3:**
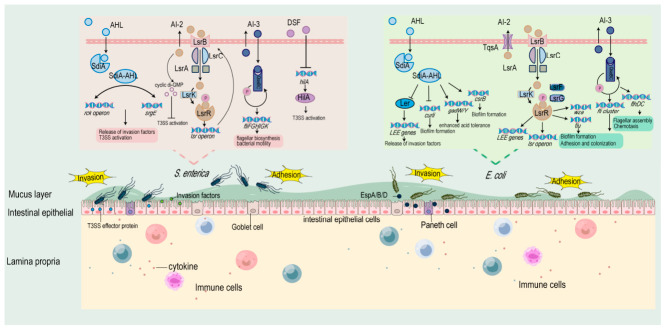
QS-regulated intestinal invasion by foodborne pathogenic bacteria.

Infection by foodborne pathogens typically begins with intestinal colonization, followed by adhesion, invasion, and virulence factor expression that disrupts host barriers and induces inflammatory responses. Accumulating evidence indicates that QS not only participates in pathogen population density sensing but also coordinates the entire infection process by integrating environmental and host-derived signals. Enteric pathogens represented by *Salmonella* and enteropathogenic *Escherichia coli* (EPEC) have established multi-level regulatory networks involving AHL, AI-2, and AI-3 signals, thereby enabling precise control over colonization, invasion, and host adaptation (Figure 3).

#### 3.2.1. QS Promotes Pathogen Colonization and Invasion

Colonization is the essential first step for foodborne pathogens to establish infection. Upon entering the host intestine, pathogens must overcome gastrointestinal environmental stresses and successfully adhere to the intestinal epithelial surface. A growing body of evidence demonstrates that QS enhances pathogen survival and colonization capacity within the host by regulating biofilm formation, flagellar motility, and the expression of invasion-associated genes.

Although *Salmonella* lacks AHL signal synthases, it encodes the LuxR homolog SdiA, which can perceive AHL signals produced by other bacteria and regulate the expression of the *rck* operon and invasion-associated genes such as *srgE*, thereby promoting cell adhesion, biofilm formation, and host invasion [45,46]. Studies have shown that *sdiA* deletion mutants exhibit phenotypes including defective biofilm formation, reduced adhesion capacity, and diminished invasion efficiency, confirming the critical role of interspecies signal perception in *Salmonella* pathogenesis [47].

Similarly, SdiA in enteropathogenic *E. coli* (EPEC) also participates in infection process regulation, albeit with pronounced stage specificity. Prior to reaching the host intestine, SdiA represses Ler (LEE-encoded regulator), the master transcriptional activator of the Locus of Enterocyte Effacement (LEE) pathogenicity island, thereby downregulating invasion factors including the adhesin intimin and secreted proteins EspA, EspB, and EspD. Simultaneously, SdiA suppresses curli gene expression to limit premature biofilm formation while activating the *gadW/Y* operon to enhance acid resistance, improving bacterial survival during gastric passage. Following successful intestinal colonization, SdiA switches to activating the *csrB* promoter, promoting expression of the small RNA CsrB to enhance biofilm formation, and concurrently represses the flagellin gene *fliC* to reduce motility [48,49,50] (Figure 3).

Beyond AHL signals, AI-2 also participates in the regulation of invasion processes. Studies have shown that *luxS* deletion in *Salmonella* leads to the downregulation of invasion-associated genes, including *sicA*, *sopB*, and *invF*, thereby impairing the type III secretion system (T3SS)-mediated host cell invasion capacity [51]. Similarly, AI-2 promotes the expression of genes within the Locus of Enterocyte Effacement (LEE) pathogenicity island in EPEC, enhancing its adhesion to and colonization of intestinal epithelial cells [52].

Notably, QS-mediated regulation of invasion does not always act in a promoting direction. AI-2 has been found to induce the accumulation of cyclic di-GMP, thereby suppressing T3SS effector protein secretion and reducing host invasion capacity [53]. Furthermore, under anaerobic conditions, exogenous AHL signal molecules can upregulate *luxS* expression [54]. These findings indicate that QS effects exhibit pronounced environmental dependence.

#### 3.2.2. QS Mediates Host Environmental Sensing and Infection Strategy Modulation

Following successful colonization, pathogens must dynamically adjust their infection strategies in response to changes in the host environment. An increasing body of evidence indicates that the QS network can integrate information from both host-derived and microbial community sources to coordinate multiple processes, including motility, virulence expression, and environmental adaptation.

Beyond population density signals, certain pathogens can also exploit QS systems such as AI-3/QseC to perceive host-derived information and adjust their infection strategies accordingly. Studies have shown that host stress-associated signals can promote flagellar motility, chemotactic behavior, and the expression of invasion-associated genes in pathogens, thereby enhancing colonization efficiency and infection success rates [55]. This environmental sensing capability enables pathogens to dynamically adjust resource allocation in response to changes in host physiological status, initiating infection programs in a concentrated manner when conditions are favorable.

Beyond host-derived signals, QS signals originating from other microorganisms can also influence pathogen virulence processes. For example, the DSF-family signal molecule cis-2-hexadecenoic acid (c2-HDA) can suppress the expression of HilA, a key transcriptional regulator in *Salmonella*, thereby reducing T3SS activity and impairing host invasion capacity [56]. This phenomenon illustrates that the pathogenic behavior of a pathogen is influenced not only by its own QS network but also by information inputs from the surrounding microbial community.

Collectively, the infection process of invasive pathogens is not fundamentally driven by a single signaling pathway but rather represents a dynamic adaptive process jointly regulated by diverse environmental cues. By integrating multidimensional information—including host status, community composition, and population density—the QS network enables pathogens to optimize infection strategies and enhance pathogenic success.

### 3.3. QS-Mediated Toxin-Type Pathogenesis

Not all foodborne diseases rely on pathogens establishing invasive infections within the host. For certain toxigenic pathogens, their pathogenic effects derive primarily from toxin production and release rather than tissue invasion per se. In this process, QS couples cell density information with virulence expression programs, enabling synchronized toxin production and concentrated release. Beyond invasive pathogenesis, therefore, QS-mediated regulation of toxigenic processes also represents an important mechanism in the occurrence of foodborne disease.

#### 3.3.1. QS Regulates Pre-Formed Toxin Accumulation in Food

Unlike infection-type food poisoning, which depends on pathogens entering and colonizing the host, the risk of toxin-type food poisoning often arises during the food storage phase. When temperature control is inadequate during processing, transportation, or storage, pathogens can proliferate within the food matrix and produce toxins. Some toxins retain activity even when bacterial numbers decline or the cells are killed during subsequent processing. In situ toxin production in food therefore constitutes a critical step in the development of toxin-type food poisoning.

**Figure 4:**
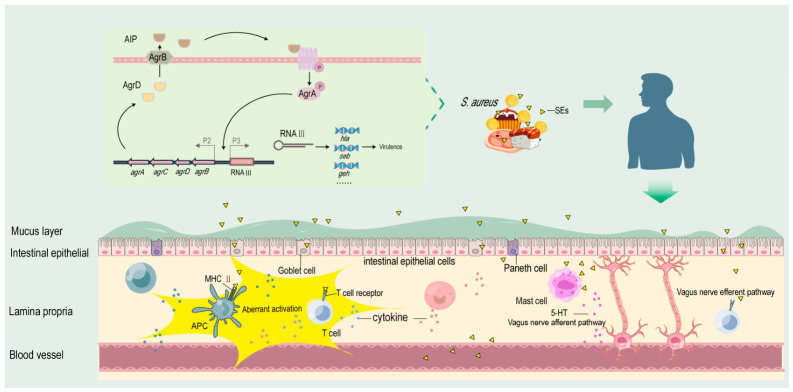
QS-mediated regulation of *Staphylococcus aureus* enterotoxin production and food poisoning.

*Staphylococcus aureus* is a prototypical pathogen responsible for in situ toxin production in food. The staphylococcal enterotoxins (SEs) it produces possess strong thermal stability and represent the principal cause of staphylococcal food poisoning [57,58,59]. Consequently, in cooked meat products, dairy products, and ready-to-eat foods, SEs accumulated during earlier stages may pose a persistent food safety risk even after viable bacterial counts have declined (Figure 4).

A growing body of evidence indicates that SE production is tightly controlled by the AIP-mediated Agr QS system. As cell density increases, extracellular AIP progressively accumulates to reach the activation threshold, whereupon the Agr signaling pathway is triggered and RNAIII expression is induced, promoting the production of SEs and other virulence factors such as α-hemolysin (Hla) [60,61]. Inhibition of AgrA activity significantly reduces the expression levels of both SEs and Hla [62], demonstrating that the Agr system performs the critical function of converting cell density information into toxin expression signals.

From an ecological perspective, this cell-density-dependent regulatory mode prevents bacteria from prematurely committing to metabolically costly toxin synthesis when the population is small. Instead, the toxigenic program is launched in a concentrated manner only after the population reaches a sufficient size, thereby enhancing toxin accumulation efficiency and pathogenic success. The QS system thus effectively serves as a critical regulatory bridge linking bacterial proliferation to in situ toxin production in food.

Similar QS-dependent toxigenic strategies have been documented in other foodborne pathogens. For example, the botulinum neurotoxin (BoNT) produced by *Clostridium botulinum* is also governed by a complex regulatory network. Current evidence suggests the possible existence of a QS regulatory mechanism analogous to the AIP-mediated system, although the cognate signal molecules and regulatory pathways await full elucidation [63]. These findings indicate that the use of QS to coordinate toxin expression may represent a shared adaptive strategy among multiple toxigenic foodborne pathogens.

Collectively, in food storage environments, QS couples the population expansion process with the toxin synthesis process, enabling pathogens to launch toxigenic programs in a concentrated manner under favorable conditions and thereby substantially elevating the risk of foodborne intoxication.

#### 3.3.2. QS Regulates Intestinal Toxin Expression and Release

Unlike pathogens such as *Staphylococcus aureus*, which produce toxins in advance during the food storage phase, certain spore-forming foodborne pathogens primarily cause disease through in situ toxin production within the intestine. These pathogens typically persist in food as spores. Following ingestion, they traverse the gastric acid barrier, enter the intestine, and germinate into vegetative cells under favorable conditions. They then produce copious amounts of exotoxins, leading to symptoms including diarrhea, vomiting, and intestinal inflammation. An increasing body of evidence indicates that QS plays a pivotal role in this process by coordinating the synchronized expression of virulence genes to achieve concentrated toxin release.

**Figure 5:**
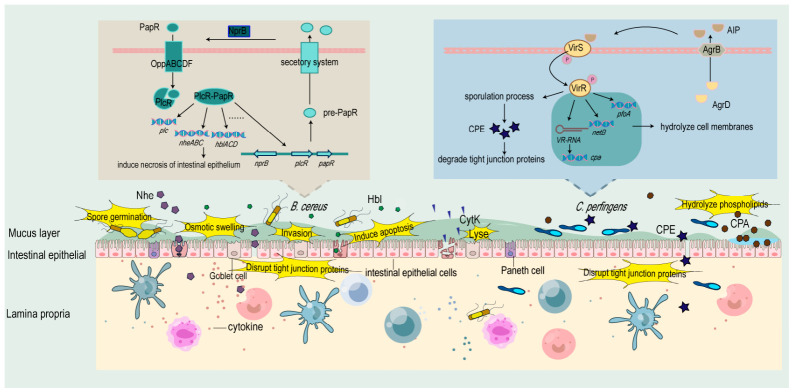
QS-mediated modulation of intestinal toxin biosynthesis in foodborne pathogenic bacteria.

*Bacillus cereus* is a prototypical foodborne pathogen that produces toxins within the intestine. Its major virulence factors responsible for diarrheal-type food poisoning include hemolysin BL (Hbl), non-hemolytic enterotoxin (Nhe), and cytotoxin K (CytK) [64,65,66]. The expression of these toxins is primarily governed by the PlcR-PapR QS system. The PapR precursor peptide is processed and released into the extracellular milieu, after which it re-enters the cell and binds to the transcriptional regulator PlcR to form an activated complex. This complex subsequently induces the expression of virulence genes including *hbl*, *nhe*, and *cytK* [67,68,69,70] (Figure 5). Studies have demonstrated that deletion of either *plcR* or *papR* significantly reduces enterotoxin production capacity [68], confirming the central role of QS in *B. cereus* virulence expression.

*Clostridium perfringens* is likewise a typical spore-forming foodborne pathogen. Upon entering the human body, its spores germinate in the small intestine, after which vegetative cells rapidly proliferate and release exotoxins, ultimately causing diarrheal-type food poisoning. Its principal virulence factors include alpha toxin (CPA), beta toxin (CPB), and *C. perfringens* enterotoxin (CPE). Among these, CPE is regarded as the most critical pathogenic determinant in human food poisoning, capable of disrupting tight junction structures of intestinal epithelial cells, leading to increased intestinal permeability and massive fluid secretion. Studies have shown that its Agr-like system regulates the expression of multiple virulence genes, including *pfoA*, *cpa*, and *netB*, through the VirS/VirR two-component system, and further amplifies the virulence response via non-coding RNAs [71,72] (Figure 5).

Although *B. cereus* and *C. perfringens* employ distinct QS regulatory modules—the former relying on the PlcR-PapR system and the latter on the Agr-like/VirS-VirR system—both exhibit a similar pathogenic logic. The pathogens first disseminate as spores and gain entry into the host intestine. During germination and proliferation, QS signals progressively accumulate. Upon reaching a threshold cell density, virulence gene expression is synchronously activated and toxins are released in a concentrated burst. Unlike invasive pathogens, which depend on sustained colonization and tissue invasion, these toxigenic pathogens achieve efficient pathogenesis within a relatively short timeframe through QS-mediated “synchronized toxigenesis,” thereby enhancing their survival and transmission advantages within the host.

## 4. QS Intervention Strategy

As discussed above, QS functions as a central regulatory network that coordinates multiple hazard-associated phenotypes in foodborne microorganisms. Given its role in controlling collective behaviors rather than bacterial viability, QS has emerged as an attractive target for food microbial control. Consequently, a variety of QS intervention strategies have been developed to disrupt signal production, transmission, or perception, thereby attenuating microbial spoilage and pathogenicity.

### 4.1. Targeting the QS Communication Network

The normal functioning of QS systems depends on signal molecule production, accumulation, recognition, and downstream regulation. Accordingly, existing QS intervention strategies largely converge on key nodes within the signal transmission process. Based on the specific step targeted, these strategies can be categorized into three classes: inhibition of signal synthesis, degradation of signal molecules, and blockade of signal recognition and transduction [73]. These strategies share the core objective of directly disrupting QS information flow, thereby attenuating bacterial collective coordination capacity and reducing virulence expression.

#### 4.1.1. Disruption of QS Signal Molecule Synthesis

**Figure 6:**
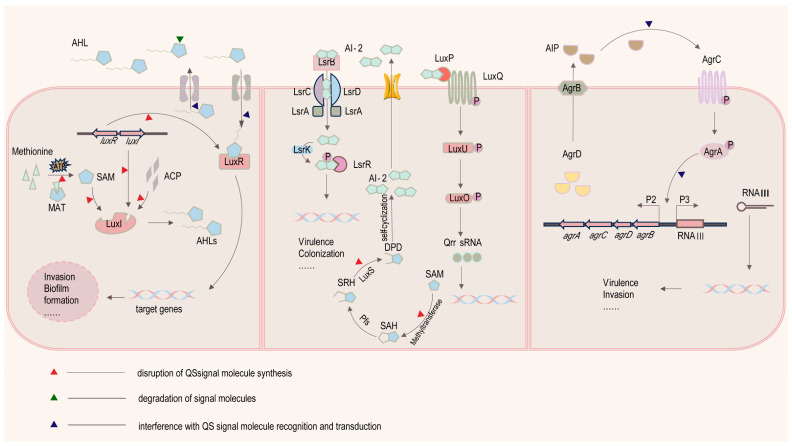
QS mediated by AHL, AI-2, and AIP and their inhibitory targets.

**Figure 7:**
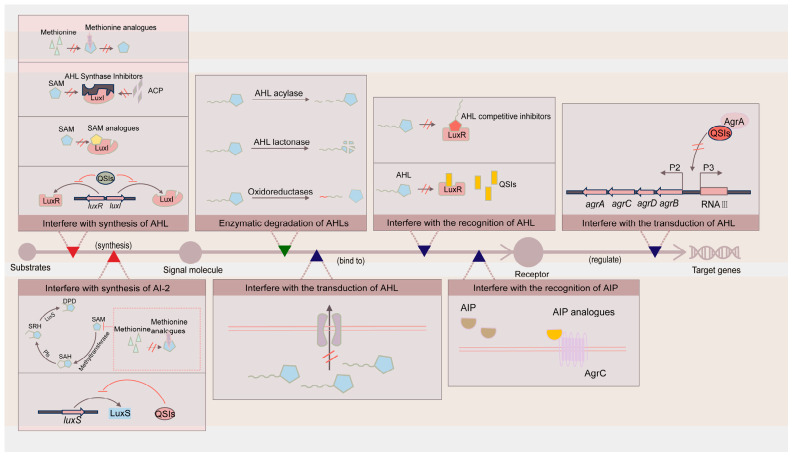
The Inhibitory Mechanisms of QS.

In AHLs synthesis, it requires the substrates SAM and ACP. SAM synthesis inhibitors (e.g., L-cycloserine) reduce AHL production by limiting substrate supply. Notably, SAM participates in the biosynthesis of both AHLs and the AI-2 precursor DPD (Figure 6 and Figure 7). Targeting SAM metabolism therefore holds promise for simultaneously disrupting intraspecies and interspecies QS communication, offering a new avenue for developing broad-spectrum QS intervention strategies. By inhibiting SAM synthesis, it is possible to disrupt both intraspecies and interspecies QS communication in bacteria. This strategy holds considerable significance for developing preservation approaches suitable for complex food matrices. In addition, SAM analogs (e.g., S-adenosylhomocysteine and 5-methylthioadenosine) competitively inhibit AHL synthase–substrate binding, suppressing signal molecule generation (Figure 6 and Figure 7) [73,74].

Beyond substrate limitations, certain intervention strategies can directly target signal molecule synthases or their encoding genes. The effector protein Le1288 secreted by *Lysobacter enzymogenes* OH11 binds to the AHL synthase PcoI of *Pseudomonas fluorescens*, blocking its interaction with the substrate and thereby inhibiting AHL biosynthesis [75]. The fungal-derived enzyme complex Flavourzyme, which is widely used in the food industry, can downregulate *luxS* expression, reducing AI-2 production levels in *Escherichia coli* and *Salmonella* [76]. In addition, certain nanomaterials have also demonstrated the potential to modulate QS signal synthesis. For example, cellulose nanocrystal/zinc oxide nanocomposites (CNCs/ZnO) can downregulate *agrB* expression in *Clostridium perfringens*, while selenium nanoparticles (SeNPs) can inhibit *luxS* expression in *E. coli*, thereby attenuating biofilm formation capacity [77,78].

#### 4.1.2. Degradation of Signal Molecules

The most extensively studied QS quenching strategies to date are those based on signal molecule inactivation mediated by AHL-degrading enzymes, which can be broadly classified into three categories: acylases, lactonases, and oxidoreductases (Figure 7) [75]. The acylase PF2571 secreted by *P*. *fluorescens* can degrade AHLs of various chain lengths. When this enzyme is heterologously expressed in *E. coli*, it significantly inhibits the QS activity of *A. veronii*, thereby delaying the spoilage process during the refrigerated storage of red snapper [79]. The lactonase AiiA from *Bacillus* spp. hydrolyzes the lactone ring of AHLs, effectively reducing the mortality rate of *Aeromonas hydrophila* infection in zebrafish [80]. Oxidoreductases, represented by CYP102A1 from *Bacillus megaterium*, achieve AHL signal inactivation by modifying the acyl side chain [81].

#### 4.1.3. Interference with QS Signal Molecule Recognition and Transduction

The signal recognition and transduction stage likewise represents an important target for QS intervention. Key mechanisms include competitive or non-competitive blockade of receptor activation and disruption of downstream signal transduction processes (Figure 6 and Figure 7). These mechanisms form the core rationale for current QS inhibitor development (Table 1). In terms of competitive inhibition, various analogs of AHLs have been developed through structural modification (such as lactone ring oxidation or acyl chain substitution). These molecules can block natural AHL signal transduction by occupying the ligand-binding domain of LuxR family receptors (Figure 7) [82]. For example, methyl p-aminobenzoate, naturally present in grapes and strawberries, can competitively bind to the LuxR receptor of *Aeromonas sobria*, inhibiting its interaction with C4-HSL. This leads to a significant reduction in biofilm formation, motility, and protease activity [83]. Similar strategies have also been applied to Gram-positive bacteria. In the *S. aureus* Agr system, AIP analogs mimic the conformation of natural signal molecules to bind to the AgrC receptor, inhibiting its autophosphorylation and downstream RNAIII activation, thereby attenuating the expression of virulence genes [84,85]. In *B. cereus*, C-terminal phosphate-modified PapR derivatives effectively inhibit the activity of the PlcR regulon [70].

Beyond artificially designed receptor antagonists, QS interference based on signal competition also occurs widely in natural microbial communities. AIPs produced by different bacterial species exhibit substantial structural diversity. Even within the genus *Staphylococcus*, AIPs generated by different strains can mutually inhibit one another through competitive binding to the AgrC receptor [86,87]. Leveraging this mechanism, researchers have explored the use of AIPs produced by harmless commensal bacteria to interfere with the *S. aureus* Agr system and suppress its virulence expression [88].

**Table 1:** Research progress in the development of QS Inhibitors.

Name	Source	Configuration	Mechanism	Targeted Bacterial	References
-	synthetic	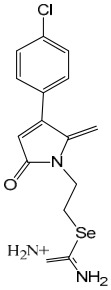	Competitively inhibit the binding of AHL to its receptor.	*P*. *aeruginosa*	[89]
-		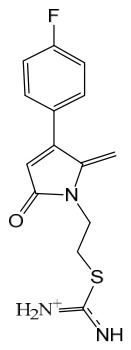		*P*. *aeruginosa*	
-	synthetic	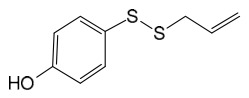	Compete for the ligand-binding site of the PQS receptor PqsR to competitively inhibit QS; down-regulate the expression of QS-related genes *lasI*, *lasR*, *rhlI*, *rhlR*, *pqsA*, and *pqsR*.		[90]
Cannabidiol	plant	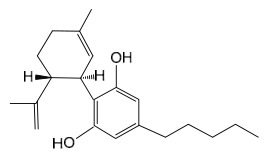	Inhibit the production of AI-2.	*S*. *aureus*	[91]
Moracin M		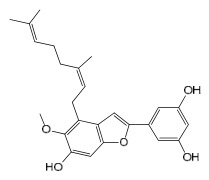			
Aspesterols A	microorganism	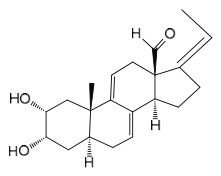	Downregulate the QS-related genes.	*P*. *aeruginosa*	[92]
Aspesterols B		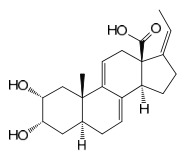			
-	synthetic	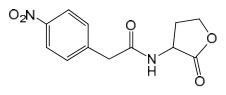	Competitively inhibit the binding of AHL to its receptor.	*P*. *aeruginosa*	[93]
-		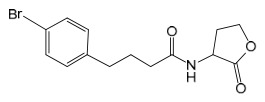			
Furanone C-30	synthetic	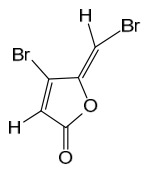	Competitively inhibit the binding of AHL to its receptor.	*P*. *aeruginosa*	[94,95]
-	synthetic	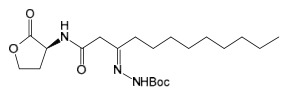	Competitively inhibit the binding of AHL to its receptor.	*P*. *aeruginosa*	[96]
-		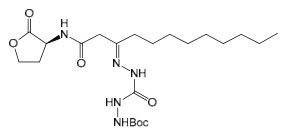			
-		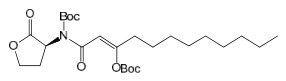			
-		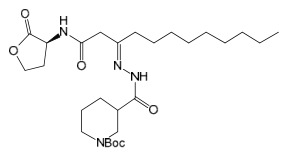			
Bnc3	synthetic	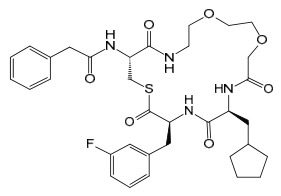	Competitive inhibition with the AIP receptor.	*S*. *aureus*	[97]
Str7410	synthetic	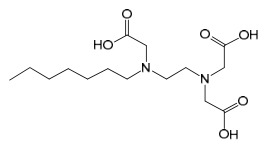	Inhibit the interspecies QS of *P. aeruginosa* by suppressing AI-2 sensing and down- regulating the expression of QS- related genes.	A co-culture system of *S*. *aureus* and *P*. *aeruginosa*	[98]
LrsL	microorganism	-	AHL quenching enzyme.	Degrade C4-HSL, C6-HSL and 3-oxo-C12-HSL	[99]
coumarin	plant	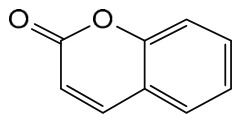	Competitively inhibit the binding of AHL to its receptor.	*P*. *aeruginosa*	[100,101]
Naringenin	plant	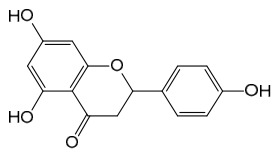	Competitively inhibit the binding of AHL to its receptor.	*P*. *aeruginosa*	[102]
TMF	plant	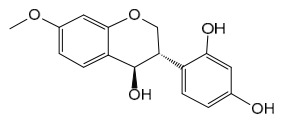	Downregulate QS-related genes; bind to the AHL receptor	*P*. *aeruginosa*	[103]
Cinnamohydroxamic acid	Synthetic	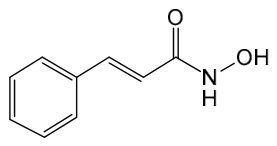	Interact with the AHL receptor RhlR; inhibit the expression of QS-related genes.	*P*. *aeruginosa*	[104]
3-Methoxy-cinnamohydroxamic acid		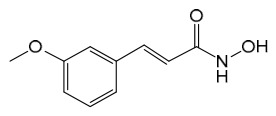			
3-Hydroxybenzoic Acid	plant	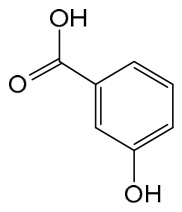	Binds to the *S. aureus* regulator AgrA and blocks signal transduction.	*S*. *aureus*	[105]
ortho-vanillin	plant	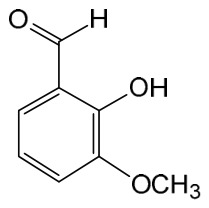	Non-competitively inhibits LasR-AHL; competitively inhibits RhlR-AHL.	*P*. *aeruginosa*	[106]
Synerazol	microorganism	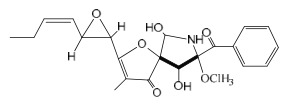	-	*S*. *aureus*	[107]
Norlobaridone	*Parmotrema*	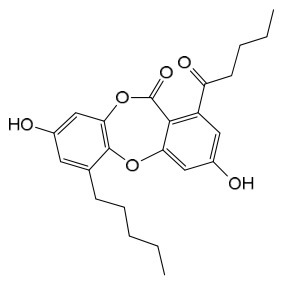	Competitively inhibit the binding of AHL to its receptor.	*P*. *aeruginosa*	[108]
l-Tyr-l-Pro	*Penicillium chrysogenum* DXY-1	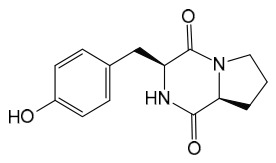	Competitively inhibit the binding of AHL to its receptor.	*P*. *aeruginosa*	[109]
citrinin	microorganism	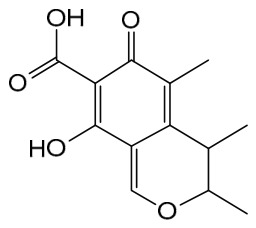	Competitively inhibit the binding of AHL to its receptor.	*P*. *aeruginosa*	[110]
Juglone	plant	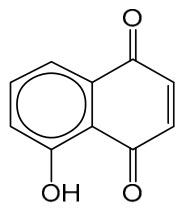	-	*P*. *aeruginosa*	[111]
-	synthetic	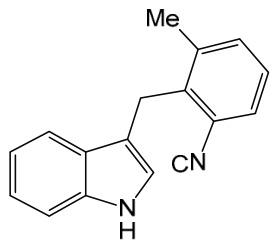	Downregulate QS-related genes and block the binding sites of C4-AHL.	*P*. *aeruginosa*	[112]
Harmine	plant	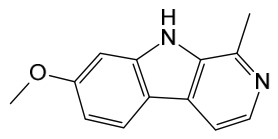	Competitively inhibit the binding of AHL to its receptor.	*P*. *aeruginosa*	[113]
Eugenol	plant	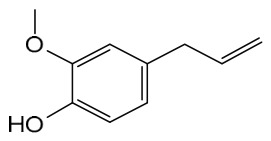	Competitively inhibit the binding of AHL to its receptor.	*E*. *coli*; *P*. *aeruginosa*; *Proteus mirabilis*; *Serratia marcescens*	[114]
Phenethyl Alcohol	plant	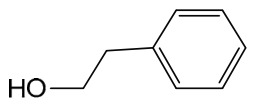	Competitively inhibit the binding of AHL to its receptor.	*Chromobacterium violaceum*; *P*. *aeruginosa*	
Kumudine B	plant	-	Competitively inhibit the binding of AHL to its receptor.	*Chromobacterium violaceum*	[115]
Cyclopentadecanone, 2-hydroxy	plant	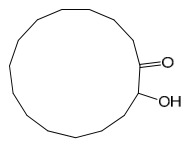	Competitively inhibit the binding of AHL to its receptor.	*Pectobacterium carotovorum* subsp. *carotovorum*	[116]
Demethylincisterol A_3_	microorganism	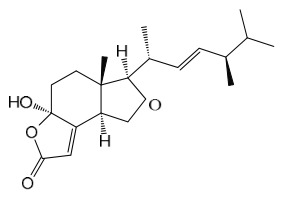	Downregulate QS-related genes.	*B*. *cereus*	[117]
-	synthetic	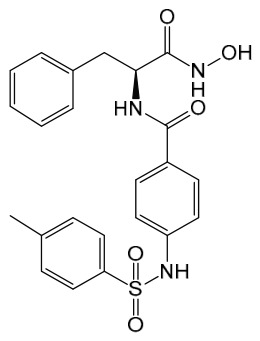	Competitively inhibit the binding of AHL to its receptor.	*P*. *aeruginosa*	[118]
-	synthetic	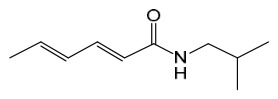	Downregulate QS-related genes; exert an inhibitory effect by binding to PqsR.	*P*. *aeruginosa*	[119]
-		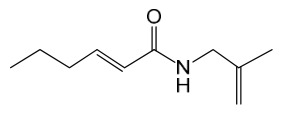			
Phenyltetrazole sulfoxide Derivatives	synthetic	-	Competitively inhibit the binding of AHL to its receptor.	*P*. *aeruginosa*	[120]
-	synthetic	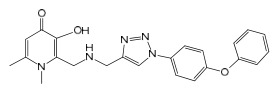	Competitively inhibit the binding of AHL to its receptor.	*P*. *aeruginosa*	[121]
Emodin	microorganism	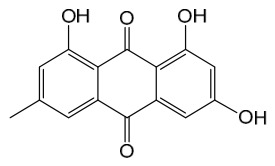	-	*Pectobacterium carotovorum* subsp. *carotovorum*	[122]
Coniochaetone K	microorganism	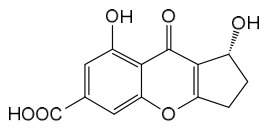	Competitively inhibit the binding of AHL to its receptor.	*Chromobacterium violaceum*	[123]
-	synthetic	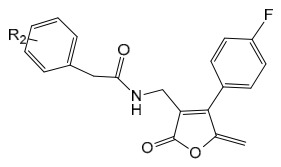	Interacts with LasR, PqsR and RhlR	*P*. *aeruginosa*	[124]
-	synthetic	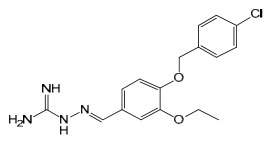	Bind to the active site of LsrK.	*Salmonella Typhimurium*; *E*. *coli*	[125]
N-(2-and 3-Pyridinyl) benzamide Derivatives	synthetic	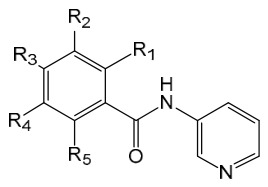	Competitively inhibit the binding of AHL to its receptor.	*P*. *aeruginosa*	[126]
*-*	synthetic	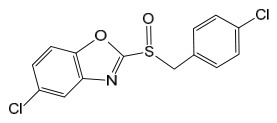	It competes with QS signal molecules for partial binding sites on the LasR receptor protein by forming hydrogen bonds.	*P*. *aeruginosa*	[127]
Curcumin	plant	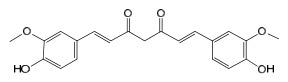	Inhibit the expression of the *luxS* gene; interact strongly with LasR and LasI.	*P*. *aeruginosa*; *B*. *subtilis*; *Streptococcus* spp.	[128,129,130]
10-undecenoic acid	plant	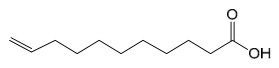	Interacts strongly with LuxS and LasI	*P*. *aeruginosa*; *B*. *subtilis*	[130]

Non-competitive inhibition is achieved by altering receptor conformation or interfering with the stability of the signal transduction complex. The flavonoid quercetin can bind to the AHL-type QS receptor proteins LasR/RhlR of *P. aeruginosa*, inducing protein structural rearrangement and loss of DNA-binding ability, thereby inhibiting QS [131]. Sodium citrate can interact with the active sites of the AHL-type QS receptor protein SmaR of *Serratia marcescens* and the LasR receptor of *P. aeruginosa*, modulating their transcriptional activity to inhibit QS [132,133]. Farnesol inhibits QS by binding to the AIP-type receptor ComP, thereby suppressing biofilm formation [134].

Intervention in signal transduction processes also holds significant potential. Small molecules such as eugenol inhibit the phosphorylation of the cytoplasmic response regulator AgrA in *S. aureus*, blocking its binding to target DNA, leading to downregulation of SEs and Hla expression [135,136]. Rootin interferes with the interaction between the DNA-binding domain of *S. aureus* AgrA and the P3 promoter, inhibiting the transcriptional activation of virulence factors such as hemolysin and protein A [137]. Astragaloside IV, eugenol, and baicalin impact the efflux pumps of *P. aeruginosa*, inhibiting the transport of AHLs and blocking QS [138].

### 4.2. Indirect QS Regulation Strategies Based on Microbial Ecological Interactions

Given that real food systems are typically composed of complex microbial communities, researchers have recently begun to explore indirect QS regulation through ecological interactions within these communities. Certain microorganisms can interfere with the QS systems of pathogens by secreting signal antagonists, metabolites, or competitive signal molecules, thereby suppressing virulence expression and biofilm formation. This microecological regulation-based strategy does not require directly targeting the pathogen itself and is therefore considered to possess favorable ecological compatibility and application potential. Tryptophan derivatives secreted by Kluyveromyces marxianus can interfere with the QS signaling pathways of *P*. *aeruginosa* and *Salmonella* [139]. Extracts from *Lactobacillus plantarum* downregulate the expression of the flagellar gene (*flaA*), hemolysin gene (*toxR*), and QS gene (*luxS*) in *V*. *parahaemolyticus* [140]. These studies demonstrate that beneficial microorganisms and their metabolites can not only directly inhibit pathogen growth but also achieve indirect QS regulation by reshaping community signaling networks, offering new avenues for ecologically friendly intervention strategies in food systems.

## 5. Advantages, Limitations, and Translational Challenges of Quorum Sensing-Targeted Interventions

### 5.1. Advantages and Application Prospects of QS-Targeted Interventions

Compared with conventional antimicrobial approaches, QS-targeted intervention strategies offer several distinct advantages for controlling food spoilage and foodborne pathogens. QS interference does not directly inhibit bacterial growth or cause cell death. Instead, it primarily attenuates the coordinated expression of virulence factors, extracellular enzymes, biofilms, and other population-density-dependent phenotypes. This antivirulence mode imposes lower selective pressure on microorganisms and is therefore theoretically less likely to induce the development of resistance.

Another important advantage lies in the fact that QS regulates multiple processes involved in both food spoilage and pathogenesis. QS participates in the regulation of numerous key phenotypes across both spoilage and pathogenic processes, including extracellular enzyme secretion, biofilm formation, adhesion and invasion, and toxin expression. Targeting QS may therefore simultaneously suppress spoilage activity and pathogenic traits without substantially compromising overall microbial viability—a feature that is particularly attractive in food systems where complete microbial elimination is neither realistic nor desirable.

Furthermore, QS-targeted interventions are highly compatible with existing preservation technologies. Studies have demonstrated that QS inhibitors can act synergistically with antimicrobial peptides, biopreservatives, natural antimicrobial substances, and packaging materials [141,142]. Such combinatorial strategies can enhance preservation efficacy while reducing the dosage of each individual agent, thereby minimizing potential impacts on sensory quality and production costs.

Nevertheless, despite these compelling advantages, successful translation of QS-targeted strategies from laboratory research to real food systems faces considerable challenges. Signal system specificity, microbial adaptability, ecological consequences, and industrial implementation constraints may all influence intervention efficacy.

### 5.2. QS Signal System Specificity Limits the Universality of Targeted QS Interventions

A major challenge facing QS-targeted interventions lies in the remarkable diversity of signal systems among food-associated microorganisms. Unlike conventional antimicrobial targets, QS regulation is not governed by a universal signaling pathway. Different bacteria employ distinct signal systems—including AHLs, AI-2, AIPs, DSF, and PQS—and inhibitors consequently tend to exhibit pronounced target specificity. For example, AHL-targeted inhibitors and quorum-quenching enzymes, while effective against many Gram-negative spoilage organisms, generally fail to interfere with Gram-positive pathogens that rely on AIP-mediated communication [12,143]. Similarly, although AI-2 is often regarded as a universal interspecies signal, its synthesis, perception mechanisms, and physiological functions vary considerably across bacterial taxa [31,144]. Disrupting AI-2 signaling may therefore fail to produce consistent effects across different microbial communities.

Beyond differences in signal chemistry, the biological functions controlled by QS are also highly species-dependent. In many foodborne pathogens, QS positively regulates biofilm formation, extracellular enzyme secretion, motility, and virulence expression. However, the regulatory consequences of QS activation are not universally conserved. For instance, in *Lysobacter brunescens* OH23, AHL signaling negatively regulates sucrose uptake and metabolism [44], whereas in *Gluconacetobacter xylinus*, enhanced QS activity promotes glucose utilization and growth [145]. These contrasting observations demonstrate that identical QS signals can elicit fundamentally different physiological outcomes in different species. Inhibiting QS may therefore not always yield a predictable reduction in spoilage potential or pathogenicity.

This challenge is compounded in real food systems, where microbial communities typically consist of multiple interacting species. Distinct QS circuits may coexist within the same ecological niche, jointly governing community behavior through signal exchange, competition, or functional redundancy. Under such conditions, targeting a single signaling pathway may suppress only a subset of the microbial population, leaving other communication networks unaffected.

Future QS intervention strategies are therefore likely to move toward scenario-specific and precision-designed approaches. For food spoilage systems dominated by a single predominant species, highly targeted signal inhibitors may be employed. For complex mixed communities, however, synergistic interventions combining multi-target inhibitors, quorum-quenching enzymes, or microecological regulation strategies will be required.

### 5.3. Ecological Impact on Beneficial Microbial Communities and Intestinal Microecological Stability

Although QS-targeted interventions are often considered more selective than conventional antimicrobial treatments, their ecological consequences remain insufficiently understood. A fundamental concern is that QS signaling is not confined to spoilage organisms and foodborne pathogens. Many beneficial microorganisms likewise rely on QS systems to coordinate population-level behaviors, including biofilm formation, stress adaptation, metabolite production, and community assembly. Interfering with QS signaling may therefore inadvertently affect non-target microbial populations.

This issue is particularly pronounced in fermented foods, where microbial succession and metabolic activities are critical for product quality and safety. Beneficial microorganisms such as lactic acid bacteria frequently employ QS-associated signaling pathways to regulate environmental adaptation, bacteriocin production, and interspecies interactions [47]. Broad-spectrum QS inhibitors may therefore alter microbial community composition or disrupt desirable fermentation processes. Although several studies have demonstrated that QS inhibitors can suppress spoilage organisms or pathogens, their effects on food-associated beneficial microbial communities have received little attention.

As a ubiquitous interspecies communication signal, AI-2 may exert interference effects that extend beyond the target strain itself, further influencing community structure and ecological interactions. However, such community-level ecological consequences remain poorly evaluated in complex food systems. An additional concern is the unknown impact of QS inhibitors on the human gut microbiota. Following food ingestion, residual QS inhibitors, quorum-quenching enzymes, or microbial metabolites may enter the gastrointestinal tract and interact with resident microbial communities. Given that QS-associated signals have been implicated in microbial colonization, biofilm development, and interspecies communication within the gut ecosystem [146], unintended interference with these processes could theoretically compromise microecological stability and host–microbe interactions.

Overall, the ecological safety of QS interventions cannot be readily assumed. Future research should move beyond single-strain models and incorporate community-level analyses, metagenomic approaches, and gut microbiome assessments to comprehensively evaluate the ecological consequences of QS-targeted interventions in food systems.

### 5.4. Industrial Scalability and Economic Feasibility

Despite the growing number of QS-targeted interventions reported in laboratory studies, their translation into commercial food preservation technologies remains very limited. Inhibitory effects achieved under laboratory conditions often prove difficult to replicate directly in real food systems.

One important challenge is the stability of QS inhibitors within food matrices. Many quorum-quenching enzymes and natural QS inhibitors are sensitive to environmental factors such as pH, temperature, ionic strength, and interactions with food components [147,148]. These factors may reduce biological activity during processing, storage, or distribution, thereby limiting practical efficacy.

Production cost also constitutes a significant barrier to large-scale application. Many natural QS inhibitors require extraction and purification from plant materials, resulting in relatively low yields and high manufacturing costs. Similarly, enzyme-based interventions often involve processes including fermentation, purification, stabilization, and formulation, which add to production expenses.

Beyond manufacturing costs, regulatory approval and consumer acceptance must also be taken into account. Many synthetic QS inhibitors remain at early stages of development and often lack comprehensive toxicological assessment. Even for naturally sourced compounds, regulatory constraints may restrict their direct application in food. In some cases, regulatory considerations associated with food additives and flavoring agents may further complicate the direct application of certain QS inhibitors in food products.

Another critical issue is the cost–benefit balance. Compared with well-established preservation methods such as refrigeration, modified atmosphere packaging, organic acids, and biopreservatives, QS-targeted interventions must demonstrate clear economic advantages or provide unique functional benefits to justify their industrial application. Future development should therefore focus not only on improving inhibitory efficacy but also on enhancing stability, reducing production costs, simplifying formulation processes, and integrating QS-targeted strategies with existing preservation technologies.

Overall, the industrial translation of QS-targeted interventions requires simultaneous consideration of biological efficacy, manufacturing feasibility, regulatory compliance, and economic sustainability.

### 5.5. Long-Term Robustness and Potential Microbial Adaptation

Unlike conventional antimicrobial agents, QS-targeted intervention strategies primarily attenuate phenotypes associated with spoilage and virulence rather than directly inhibiting bacterial growth. This mode of action is generally considered to impose lower selective pressure on microbial populations and is therefore regarded as a promising strategy for sustainable microbial control in food systems.

However, microorganisms possess highly flexible regulatory networks that enable them to respond to changing environmental conditions. In certain cases, disruption of QS signaling may be partially compensated by alternative regulatory pathways or intercellular communication mechanisms [149]. Such regulatory plasticity may influence the strength and durability of QS inhibition across different ecological contexts, particularly within complex food-associated microbial communities.

Although current evidence generally supports the view that QSIs exert lower selective pressure than conventional antimicrobials, most available studies have focused on short-term responses, whereas the evolutionary consequences of prolonged QS disruption remain insufficiently explored. Experimental observations also suggest that adaptation to quorum-sensing interference is possible. For example, *P*. *aeruginosa* has been reported to exhibit reduced susceptibility to the quorum-sensing inhibitor brominated furanone C-30 through enhanced efflux activity, indicating that resistance-like responses may emerge under certain conditions [150].

Despite these uncertainties, current evidence does not suggest that microbial adaptation will inevitably compromise the effectiveness of QS-targeted interventions. Rather, the available findings highlight the need to evaluate QSI performance within an ecological and evolutionary framework. Future studies should therefore move beyond short-term laboratory assessments and examine the long-term stability of QS disruption under conditions that more closely resemble food processing, storage, and multispecies microbial communities. Such investigations will be essential for determining the durability and practical applicability of QS-based control strategies in real food systems.

## 6. Conclusions

This review focuses on the role of QS in food contamination and foodborne diseases. Based on the current situation of food contamination and the diverse pathogenic mechanisms of foodborne pathogens, it systematically demonstrates the central position of QS in food spoilage and the occurrence of foodborne diseases, establishing QS as a key target for food preservation and the prevention of foodborne diseases. It also summarizes the research progress on QS inhibitors.

Recent studies have revealed that the involvement of QS in food spoilage is far greater than previously expected. QS is well-known for its roles in biofilm formation on food surfaces and chemotaxis. In addition, the regulatory mechanisms of QS on the synthesis of hydrolases and secondary metabolites such as cadaverine and putrescine are gradually coming into the research spotlight. These new findings further highlight the importance of QS in food spoilage. As research deepens, more food contamination phenomena regulated or participated in by microbial QS will be discovered. The scope of food contamination phenotypes will expand from the production of spoilage substances to aspects such as texture changes and nutrient degradation, and the relationship between QS and food contamination will become clearer.

For foodborne diseases, QS functionality now extends beyond interspecies communication. Current research focuses more on in-depth exploration of the cascade regulatory mechanisms of multiple signal molecules within a species, the differential regulatory patterns of the same signal molecule among different bacterial species, and the potential complex interactions between pathogens and hosts. At present, our understanding of QS mechanisms is still far from in-depth and comprehensive. In recent years, some unconventional regulatory relationships and new complex mechanisms, such as the interaction between microorganisms and hosts (the colonization of A. hydrophila activates the host cell apoptosis program), the bidirectional effect of AI-2 in *Salmonella*, the cross-talk between QS systems and environmental signals, and the inter-species competition between *E. coli* and *Salmonella mediated* by AI-2, have provided a new perspective for in-depth understanding of the relationships between microorganisms and microorganisms, and between microorganisms and hosts. The discovery of new mechanisms theoretically increases the selection of targets for control measures.

Despite the pivotal role of QS in food spoilage and foodborne diseases, targeting QS for prevention and control faces persistent challenges. Although QS has emerged as a highly promising intervention target for food safety, its practical application remains constrained by multiple obstacles, including pronounced species specificity among distinct signal systems, dynamic reconfiguration of signaling networks within complex food matrices, potential ecological impacts of QS inhibitors on non-target microbial communities, and stability, safety, and cost control issues during scale-up. These challenges frequently involve multi-level signaling networks, dynamic environmental responses, and complex microecological interactions that are difficult to resolve comprehensively through traditional experimental approaches alone. With advances in multi-omics technologies, artificial intelligence, and machine learning, QS database construction, signal–receptor interaction prediction, community communication network modeling, and QSI virtual screening are gradually becoming feasible, offering new technical foundations for achieving precision QS-based interventions. Emerging AI technologies may facilitate the establishment of QS databases [151], while large-model simulation of signal–target binding [146], QS communication network prediction, and computer-aided QSI screening [152] represent powerful tools for addressing these challenges.

In summary, quorum sensing (QS) represents a critical target for the prevention of food spoilage and foodborne disease, and sustained deepening of research in this area carries profound significance. On one hand, it provides novel intervention paradigms for reducing food safety risks and safeguarding public health. On the other hand, it advances the implementation of precision prevention and control strategies grounded in QS mechanisms, enabling effective suppression of food spoilage and foodborne disease. From a long-term perspective, this will provide core support for building a sustainable food safety system.

## Data Availability

No new data were created or analyzed in this study. Data sharing is not applicable to this article.
